# Comparison of 3T and 7T MRI for the visualization of globus pallidus sub-segments

**DOI:** 10.1038/s41598-019-54880-x

**Published:** 2019-12-04

**Authors:** Shuki Maruyama, Masaki Fukunaga, Hans-Peter Fautz, Robin Heidemann, Norihiro Sadato

**Affiliations:** 1 Department of System Neuroscience, Division of Cerebral Integration, National Institute for Physiological Sciences (NIPS), 38 Nishigonaka, Myodaiji, Okazaki, Aichi 444-8585 Japan; 2 Department of Physiological Sciences, School of Life Science, SOKENDAI (The Graduate University for Advanced Studies), Shonan Village, Hayama, Kanagawa 240-0193 Japan; 3 Siemens Healthineers, Allee am Roethelheimpark 2, 91052 Erlangen, Germany

**Keywords:** Parkinson's disease, Brain

## Abstract

The success of deep brain stimulation (DBS) targeting the internal globus pallidus (GPi) depends on the accuracy of electrode localization inside the GPi. In this study, we sought to compare visualization of the medial medullary lamina (MML) and accessory medullary lamina (AML) between proton density-weighted (PDW) and T2-weighted (T2W) sequences on 3T and 7T MRI scanners. Eleven healthy participants (five men and six women; age, 19–28 years; mean, 21.5) and one 61-year-old man were scanned using two-dimensional turbo spin-echo PDW and T2W sequences on 3T and 7T MRI scanners with a 32-channel receiver head coil and a single-channel transmission coil. Profiles of signal intensity were obtained from the pixel values of straight lines over the GP regions crossing the MML and AML. Contrast ratios (CRs) for GPe/MML, GPie/MML, GPie/AML, and GPii/AML were calculated. Qualitatively, 7T visualized both the MML and AML, whereas 3T visualized the MML less clearly and hardly depicted the AML. The T2W sequence at 7T yielded significantly higher CRs for GPie/MML, GPie/AML, and GPii/AML than the PDW sequence at 7T or 3T. The T2W sequence at 7T allows visualization of the internal structures of GPi segments with high signal intensity and contrast.

## Introduction

Deep brain stimulation (DBS) is a stereotactic neurosurgical technique involving the placement of stimulating electrodes to the small subcortical structure^1^. DBS targeting the internal region of Globus Pallidus (GPi-DBS) is the treatment of choice for later stages of Parkinson’s disease and medical refractory generalized and segmented dystonia^2^. The clinical efficiency of GPi-DBS depends on accurate localization of electrodes inside the GPi^3–5^.

The GPi is surrounded with the external GP (GPe) and putamen anteriorly, posteriorly, and laterally, the internal capsule (CI), zona incerta (ZI) and medial forebrain bundle (MFB) medially, the nucleus of ansa lenticularis mediodorsally, the optical tract (OPT) ventrally, the amygdala laterodorsally, and the ventral GP laterodorsally^6^. Electrical current may spread into these regions. Thus the proper placement of the electrode and control of electrical current is critical to prevent side effects^2^. More importantly, the stimulation of distinct regions within the GPi causes a different therapeutic outcome. For example, stimulation of the dorsal region of the GPi improves signs and symptoms associated with Parkinson’s disease such as hypokinesia and rigidity. By contrast, although stimulation of the posteroventral region of the GPi reduces hyperkinesia induced by increasing the levodopa dose, it may aggravate gait hypokinesia^2,3,7^.

Although the mechanism of the effectiveness of DBS is still incompletely understood, it is supposed to inhibit or excite local neuronal elements^8^. There are two theories of improving movement disorders by stimulation: one is based on the function similar to disease (inhibition)^9^; the other is the fact that high-frequency stimulation excites local neuronal elements as local single-pulse stimulation (excitation). This mechanism may include abnormal activity patterns or normalizing neuronal activity pattern^10–13^, and inhibition of output nuclei within the basal ganglia circuitry. Nambu^9^ concluded that the mechanism of stimulation of the basal ganglia might be abnormal information flow within the circuit in dyskinesia. To further reveal the mechanism of the effective GPi-DBS, detailed anatomical knowledge of the subdivision of the GPi is critical.

**Figure 1:**
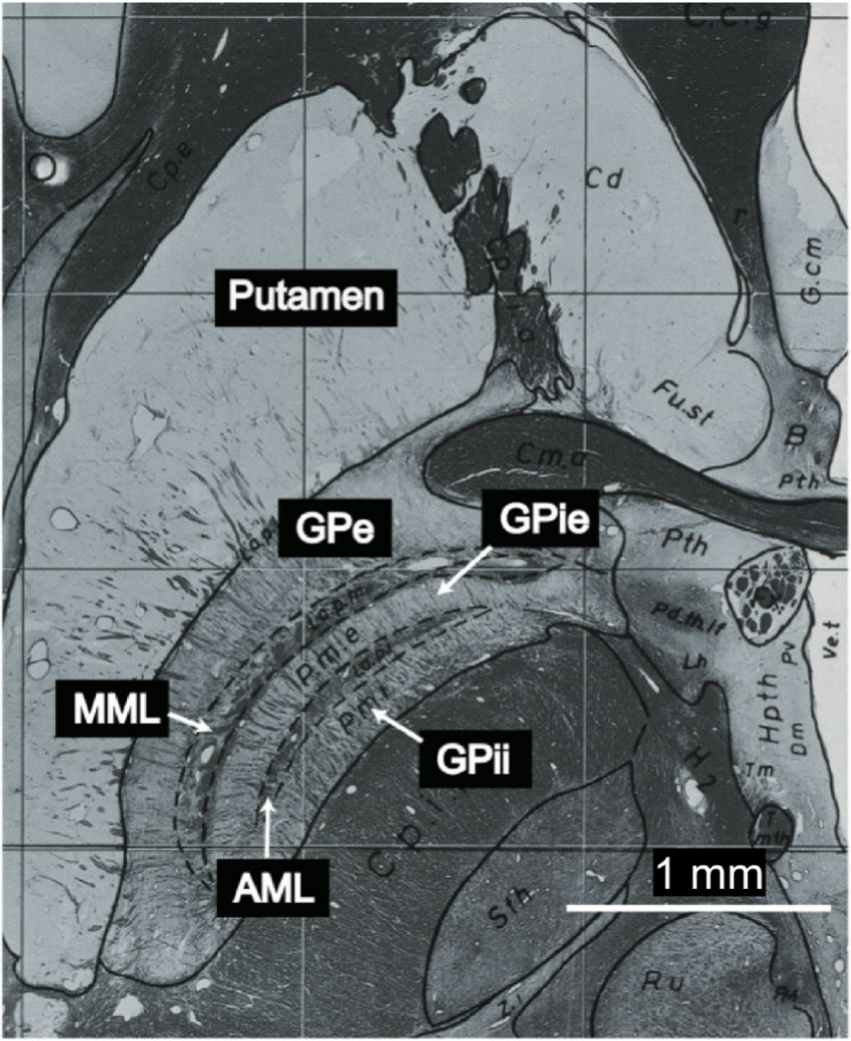
Myelin stain at the level of the GPi (plate 54) from the Schaltenbrand and Wahren atlas for stereotaxy of the human brain^16^. The images are not covered by the CC BY license. All rights reserved, used with permission from Georg Thieme Verlag KG, Germany.

The GPi, separated from the GPe by the medial medullary lamina (MML), further into the external/internal segment (GPie/GPii) by the accessory medullary lamina (AML)^8,14–16^ (Fig. 1). The localization of the GPi can be visualized pre-operatively from two-dimensional (2D) turbo spin-echo (TSE) proton density-weighted (PDW) or T2-weighted (T2W) images using magnetic resonance imaging (MRI)^17–19^. O’Gorman *et al*.^19^ reported that among various MR imaging sequences [T1-weighted (T1W), T2*-weighted (T2*W), susceptibility-weighted image (SWI), inversion recovery with TSE (IR-TSE), and phase-sensitive IR (PSIR)], the TSE PDW sequence at 1.5T achieves the best visualization of the MML. However, those authors were not always able to visualize the MML. Also, it is difficult to differentiate the GPie and GPii using conventional 1.5T or 3T MRI because the GPi segments are quite small and exhibit low contrast with the AML (Fig. 1).

Recently, ultra-high-field (static magnetic field ≥ 7T) MRI has attracted increasing attention because it can provide higher signal-to-noise-ratio, spatial resolution, and contrast than 1.5T and 3T MRI^20,21^. An increase in the static magnetic field helps visualize microstructures *in vivo* within a reasonable scan time^21–23^. Accordingly, several attempts have been made to visualize subcortical microstructures, including the GPi, at 7T^24–26^. One study identified the internal structures of the GPi (GPie, GPii, and AML) in quantitative magnetic susceptibility mapping (QSM) images using 7T MRI^23^. However, those authors utilized a method referred to as “calculation of susceptibility through multiple orientation sampling (COSMOS)”^27,28^, which took approximately 50 minutes to acquire all gradient (recalled) echo (GRE) data; consequently, this technique is not clinically feasible.

In the present study, we attempted to apply TSE sequences at 7T to obtain ultra-high-resolution images for identifying anatomical substructures of GPi segments within a clinically reasonable scan time. The TSE sequences are less susceptible to inhomogeneity of the static magnetic field than the GRE sequence. By contrast, TSE sequences are associated with several challenges, including inhomogeneity in the transmit magnetic field (B_1_^+^ field) and high specific absorption rate (SAR) of the radiofrequency (RF) pulse^29,30^. After optimization of the scan parameters such as input power, flip angle, turbo factor, and repetition time (TR) of TSE sequences within the SAR limitations^30^, we visualized the MML and AML using PDW and T2W sequences. The performance of the 7T was compared with 3T images obtained from the same participants.

## Results

### Qualitative analysis

**Figure 2:**
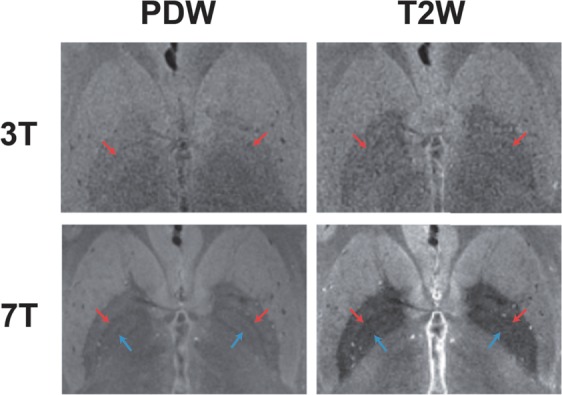
Comparison of the PDW and T2W images of the same participant at 3T and 7T. 7T visualized both the MML (red arrow) and AML (blue arrow), whereas 3T visualized the MML less clearly and hardly depicted the AML.

Figure 2 shows a comparison of PDW and T2W images taken at 3T and 7T from the same participant. The 7T image visualized both the MML (red arrow) and AML (blue arrow), whereas the 3T image visualized the MML less clearly and hardly depicted the AML as a low SI border within the GP. Specifically, the T2W image at 7T successfully visualized both the MML and AML and achieved high contrast between the GP and surrounding tissues (putamen and internal capsule).

Table 1 summarizes our qualitative analysis of the visibility of the MML and the AML. The 7T image almost entirely visualized both the MML and AML. The visibility of AML was less than 10% at 3T but greater than 90% at 7T.

**Table 1:** Qualitative analysis of the visibility of the MML and AML.

		MML		AML	
		Left	Right	Left	Right
7T	PDW	10 (90.9%)	11 (100%)	10 (90.9%)	10 (90.9%)
	T2W	11 (100%)	10 (90.9%)	10 (90.9%)	10 (90.9%)
3T	PDW	5 (45.5%)	8 (72.7%)	1 (9.1%)	1 (9.1%)
	T2W	4 (36.4%)	4 (36.4%)	0 (0%)	1 (9.1%)

### Quantitative analysis

**Figure 3:**
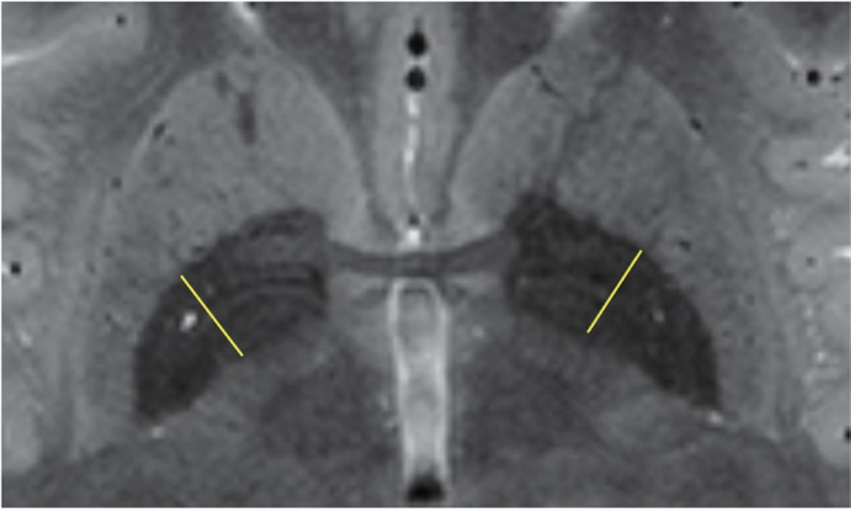
A typical example of an SI map acquired with the T2W sequence at 7T, with typical profile positions (yellow line).

**Figure 4:**
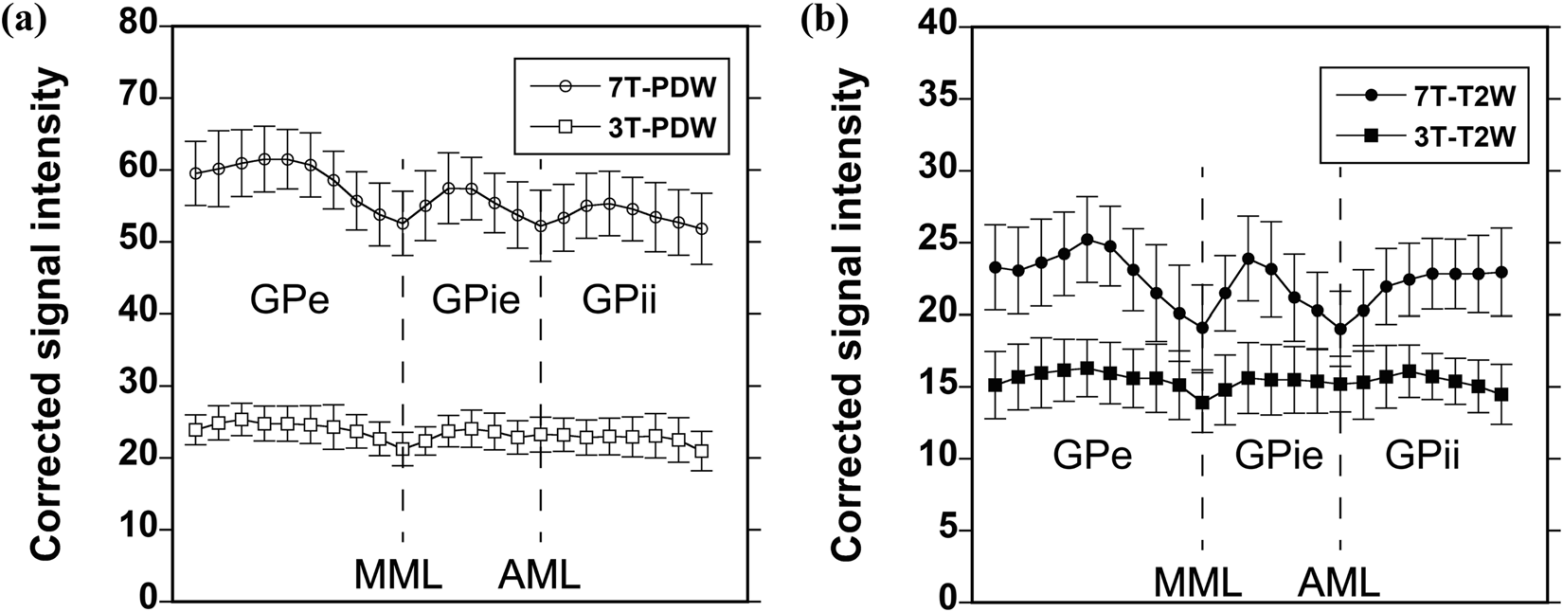
Comparison of SI profiles for the GP region acquired with the PDW (**a**) and T2W (**b**) sequences at 3T and 7T. Data are means ± standard deviation (n = 11).

Figure 3 shows a typical example of the signal intensity (SI) map with a T2W sequence at 7T and profile positions (yellow line). Figure 4(a) shows a comparison of the SI profiles for the GP region acquired with the PDW sequence at 3T and 7T, and Fig. 4(b) shows a comparison of the SI profiles for the GP region acquired with T2W sequence at both field strengths. The PDW sequence at 7T provided the highest SI. The PDW sequence at 7T exhibited an approximately 2.5-fold increase in SI relative to 3T, whereas the T2W sequence exhibited an approximately 1.6-fold increase. The SI profiles acquired with the PDW and T2W sequences at 3T contain one negative peak, representing the MML (Fig. 4(a)), whereas those acquired with the PDW and T2W sequences at 7T contain two negative peaks representing the MML and AML, distinguishing the GPe, GPie, and GPii (Fig. 4(b)).

**Figure 5:**
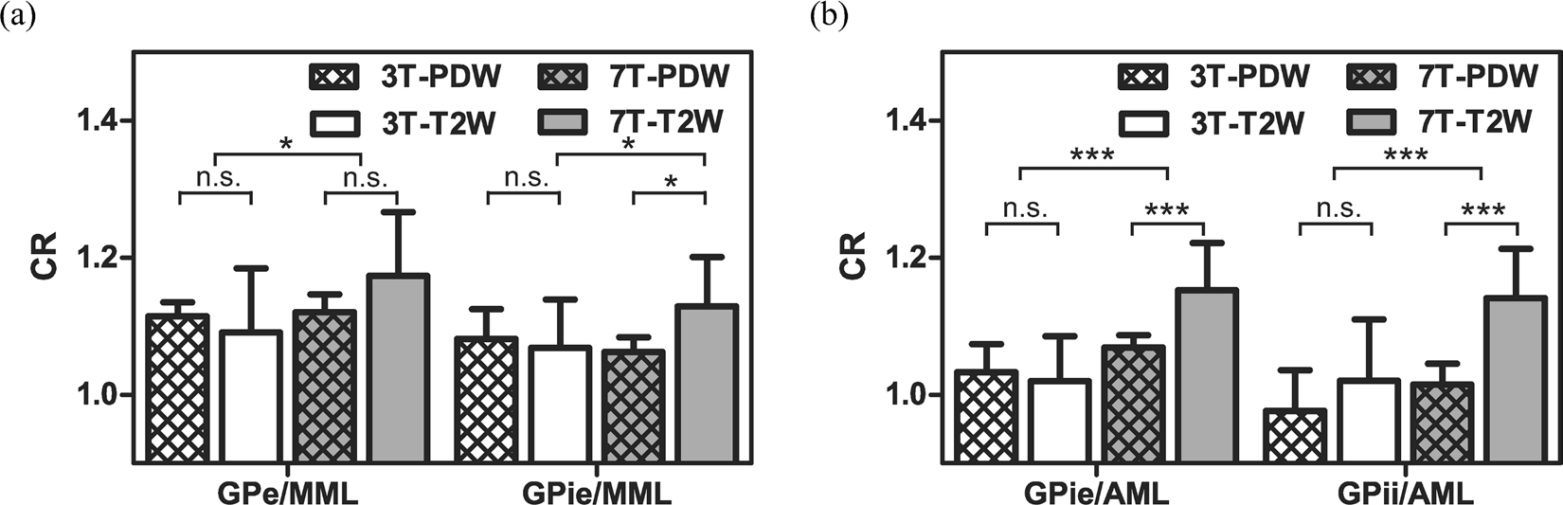
Comparison of CRs for GPe/MML, GPie/MML, GPie/AML, and GPii/AML. Data are means ± standard deviation (n = 11). Asterisks indicate the significance level of two-way ANOVA and post-hoc two-sample t-test (***P < 0.001, *P < 0.05).

Figure 5(a) shows a comparison of contrast ratios (CRs) for the GPe/MML and the GPie/MML. For the GPe/MML, the CRs were 1.11 ± 0.02 for 3T-PDW, 1.09 ± 0.09 for 3T-T2W, 1.12 ± 0.02 for 7T-PDW, and 1.17 ± 0.10 for 7T-T2W, respectively. By contrast, for the GPie/MML, the CRs were 1.08 ± 0.04 for 3T-PDW, 1.07 ± 0.07 for 3T-T2W, 1.06 ± 0.03 for 7T-PDW, and 1.12 ± 0.07 for 7T-T2W, respectively. Although there was a significant main effect of static magnetic field in GPe/MML (*F* = 4.644, *P* = 0.037), no such effect was observed in GPie/MML (*F* = 1.529, *P* > 0.1). No significant main effect of sequence was observed in GPe/MML, and GPie/MML (*F* = 0.278, *P* > 0.1; *F* = 2.289, *P* > 0.1). However, there was a interaction effect between static magnetic field and sequence in GPie/MML (*F* = 5.263, *P* = 0.027). Post-hoc two-sample t-test showed that CRs of 7T-T2W was significantly higher than 3T-T2W and 7T-PDW in GPie/MML (*P* = 0.017, *P* = 0.010; Bonferroni corrected). No other significant difference in CR was observed in GPe/MML or GPie/MML.

Figure 5(b) shows a comparison of the CRs for GPie/AML and GPii/AML. For the GPie/AML, the CRs were 1.03 ± 0.04 for 3T-PDW, 1.02 ± 0.07 for 3T-T2W, 1.07 ± 0.02 for 7T-PDW, and 1.15 ± 0.07 for 7T-T2W, respectively. Similar results were shown in GPii/AML; the CRs were 0.98 ± 0.06 for 3T-PDW, 1.02 ± 0.10 for 3T-T2W, 1.03 ± 0.04 for 7T-PDW, and 1.14 ± 0.07 for 7T-T2W, respectively. There were significant main effects of static magnetic field in GPie/AML (*F* = 30.680, *P < *0.001) and GPii/AML (*F* = 17.834, *P < *0.001). Also, we observed significant main effects of sequence in GPie/AML (*F* = 5.292, *P* = 0.027) and GPii/AML (*F* = 14.786, *P* < 0.001). Although the interaction effect between static magnetic field and sequence was significant in GPie/AML (*F* = 9.696, *P* = 0.003), no such effect was observed in GPii /AML (*F* = 2.943, *P* = 0.094). Post-hoc two-sample t-test revealed that the T2W sequence yielded significantly higher CRs in GPie/AML than the PDW sequence at 7T (*P* < 0.001, Bonferroni corrected).

**Figure 6:**
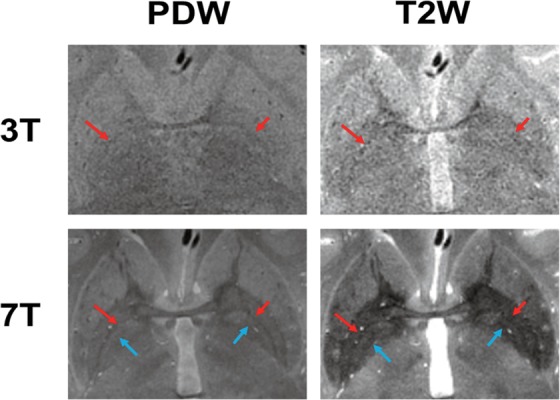
Comparison of the PDW and T2W images of the elderly participant at 3T and 7T. 7T visualized both the MML (red arrow) and AML (blue arrow), whereas 3T visualized the MML less clearly and hardly depicted the AML.

Figure 6 shows a comparison of PDW and T2W images of an elderly volunteer taken at 3T and 7T. The MML and AML were clearly visualized in the PDW and T2W images at 7T. For the elderly participant, similar results were obtained with young volunteers.

## Discussion

In this study, we quantitatively and qualitatively compared the visualization of the MML and AML between PDW and T2W sequences at 3T and 7T. Our results demonstrated that PDW and T2W sequences at 7T almost clearly visualized both the MML and the AML. By contrast, in PDW and T2W sequences at 3T, the MML was visualized to some extent, whereas the AML was barely visualized. Also, we showed that the T2W sequence visualized both the MML and AML with significantly higher CRs than the PDW sequence at 7T. To the best of our knowledge, this is the first report to demonstrate that the T2W sequence at 7T allowed the visualization of the internal structures of GPi segments with high SI and contrast.

The therapeutic efficiency of DBS for the treatment of movement disorders depends on the accurate placement of electrodes^3–5^. Since the anatomical size, position and functional segregation of the GP varies considerably across individuals^3,18^, it is clinically valuable to use 7T MRI to obtain ultra-high-resolution images that accurately identify the anatomical detail of the target before the operation. In this context, scan time is an important factor. It should be noted that we obtained ultra-high-resolution images of GPi segments using TSE PDW or T2W sequences in only 8 minutes. Although the TSE sequence can provide images without degradation in quality due to inhomogeneity of the static magnetic field, in contrast to the GRE sequence, the TSE sequence has a significant issue of B_1_^+^ inhomogeneity at 7T. To solve this problem, we used dielectric pads to improve B_1_^+^ inhomogeneity and reduced the required input power. We visually evaluated the effect of various flip angle groups (120°, 140°, 160°, and 180°) on B_1_^+^ inhomogeneity in the TSE PDW and T2W images at the slice level of the GP region. However, because we observed little difference in image inhomogeneity among the flip angle groups, we used a flip angle of 180° to obtain higher SI. The TR and turbo factor were adjusted so that all scans could be performed within the SAR limit of normal operating mode.

For DBS targeting, coronal images may also be useful. Nölte *et al*.^31^ have reported that the GPi was more clearly visualized in axial than coronal images using the T2*W sequence. When we applied the MR imaging parameters for axial to coronal scanning, substantial cerebrospinal fluid (csf) ghost artifacts significantly deteriorated the visualization of the MML and the AML. To minimize the ghost artifacts, synchronizing MRI data acquisition with the cardiac cycle of individual participants is useful^32^, which requires longer scan time, resulting in the reduction of clinical feasibility. Therefore, in this study, we acquired the axial images from all participants.

The MML within GP was completely visualized by 7T, whereas less clearly by 3T (Fig. 2 and Table 1). There was a tendency that 3T-PDW showed higher visibility of the MML and higher CRs in the GPe/MML and GPie/MML than 3T-T2W (Figs. 2, 5(a) and Table 1), suggesting that the PDW sequence would be superior to the T2W sequence for visualizing the MML at 3T. These findings are consistent with 1.5T results reported previously^19^ and are explained by the fact that the PDW sequence can provide higher SI reflecting the proton density of tissues since the PDW sequence can minimize the effects of both spin-lattice relaxation time (T1) and spin-spin relaxation time (T2) on the SI in images. Quantitatively, as shown in Fig. 5(a), there was a main effect of the static magnetic field on the contras ratio (CR) of the GPe/MML, consistent with the qualitative findings. The 7T-PDW shows trends of less CR than 3T-PDW for GPie/MML (Fig. 5a) without statistical significance. Thus it is safe to mention that the PDW sequence of 7T, compared with 3T, provides no improvement in CR. Although we observed no significant difference in CR between 7T-PDW and 7T-T2W in the GPe/MML, we did observe a significant difference in the GPie/MML, indicating that the T2W sequence of 7T has an advantage in visualizing the MML over 3T, leading to better discrimination of GPi from GPe. Since parts of the MML consist of the nerve fibers from the striatum^33^, the MML has high myelin content^16,23,34,35^. The 7T-T2W enhances the T2-shortening effects of myelin content in the MML while maintaining a higher SI in the iron-rich GP than 3T.

The AML within the GPi was almost completely visualized by 7T, but not by 3T (Fig. 2 and Table 1). The SI profiles at 7T contained discrete negative peaks corresponding to the AML, whereas no such peak was evident in 3T (Fig. 4). As shown in Fig. 5(b), the main effects of the static magnetic field on the CRs in the GPie/AML and GPii/AML were significant, indicating that 7T provided better contrast of the AML with the surrounding structures (GPie and GPii). The T2W sequence yielded significantly higher CRs in the AML than the PDW sequence at 7T, probably through the same mechanism as MML.

The present study has a few limitations. First, our participants were almost young, healthy individuals. Ide *et al*.^34^ reported that there was no significant difference in the visualization of the MML, using QSM and phase difference-enhanced imaging, between ordinary healthy people and patients with Parkinson’s disease. Also, they reported that the deposition of iron content in the GP increases with age^35^. Thus, it is possible that age-related iron deposition in the GP region will affect the visualization of the MML and AML in T2W sequences due to the shortening of the T_2_. We additionally acquired the images of an elder participant as a preliminary trial. The visibility of the MML and the AML of an elderly participant was similar to that of young participants between 7T and 3T (Fig. 6), suggesting that 7T will be superior to 3T for identifying the subdivision of GP segments regardless of age. However, due to the limited number of participants, further study will be needed to investigate the magnetic susceptibility effect due to age-related physiological iron/calcium deposition on the visualization of the MML and the AML.

Second, the present study does not precisely demonstrate the improvement of the accuracy of electrode placement within GPi, which will bring better clinical outcomes of GPi-DBS. Further clinical studies are necessary to prove the superior efficacy of 7T over 3T in DBS targeting in the clinical settings, testing the visualization of the internal structures of GP of the elderly population, and the clinical efficacy of DBS targeting guided by the identification of the localization of the AML.

In conclusion, we successfully obtained ultra-high-resolution images for identifying anatomical substructures of GPi segments using PDW and T2W sequences at 7T. Excellent visibility of the AML is useful for differentiating the GPie from the GPii, aiding the orientation for DBS.

## Methods

### Participants

This study was approved by the ethical committee of the National Institute for Physiological Sciences, Okazaki, Japan, and was conducted according to the Declaration of Helsinki’s guidelines for research involving humans. Written informed consent was obtained from all participants before participation. The participants were eleven healthy volunteers (five men and six women; age, 19–28 years [mean, 21.5]). Also, a 61-year-old man was included in this study. None of the participants had any previous history of neurological or psychiatric disorders.

### MR imaging protocol

All participants were scanned on a 3T MRI scanner (MAGNETOM Verio, Siemens Healthcare, Erlangen, Germany) with a 32-channel receive head coil (Siemens Healthineers, Erlangen, Germany) and a 7T MRI scanner (MAGNETOM 7T, Siemens Healthineers, Erlangen, Germany) with a 32-channel receive head coil and a single-channel transmit coil (Nova Medical Inc., MA, USA). We acquired 2D TSE PDW and T2W images of the whole GP in the axial direction parallel to the anterior commissure-posterior commissure line (AC-PC line) at 3T and 7T. Scan parameters for TSE sequences as follows: TR = 5000 msec; TE = 13 msec for PDW and 53 msec for T2W; matrix size = 448 × 348 at 3T and 432 × 344 at 7T; number of acquisitions (NA) = 2; turbo factor = 7; field of view (FOV) = 224 × 174 mm^2^ at 3T and 216 × 172 mm^2^ at 7T; in-plane spatial resolution = 0.5 × 0.5 mm^2^; slices = 19; slice thickness = 0.8 mm; bandwidth = 183 Hz/pixel at 3T and 161 Hz/pixel at 7T; acquisition time = 8 minutes 17 sec, and flip angle = 180°. The specific MR imaging parameters are listed in Table 2. The TE of the T2W sequence was optimized with the use of the multi-echo SE sequence and different TEs from 30 msec to 90 msec in 15 msec increments. To visualize the submillimeter microstructure like the MML and the AML, ultra-high-resolution data with 0.5 × 0.5 × 0.8 mm^3^ was shown to be necessary. Therefore, we compared the visualization of the MML and the AML using PDW and T2W sequences at the same resolution between 3T and 7T.

**Table 2:** MR Imaging parameters.

	FOV	Matrix	Resolution	Slices	TR	TE	Bandwidth	NA	Acquisition time
3T	224 × 174 (mm^2^)	448 × 348	0.5 × 0.5 × 0.8 (mm^3^)	19	5000 msec	13 msec (PDW)	183 (Hz/Pixel)	2	8 min 17 sec
7T	216 × 172 (mm^2^)	432 × 344				53 msec (T2W)	161 (Hz/Pixel)		

At 7T, both T2W and PDW image acquisitions were performed with a prototype TSE sequence, featuring modified RF-pulse shapes for SAR reduction. All scans were performed within the SAR limit of normal operation mode. Dielectric pads were placed to the right and left sides of the participant’s head while scanning at 7T to improve the uniformity of image intensity resulting from B_1_^+^ field inhomogeneity^36,37^. A B_1_^+^ map in the center of the brain at the slice containing the GP region was acquired for each participant in order to optimize input power and accurately produce a 90° pulse for TSE sequences. To reduce motion artifacts in the images, which would diminish the visibility of the MML and AML, we collected k-space lines randomly in the segments of the TSE sequences.

### Image analysis

For all image analyses, we selected a single slice in which the MML and AML could most easily be identified for right and left GPs. As described above, we acquired all axial images parallel to the AC-PC line at 3T and 7T. Nonetheless, because the slice levels acquired at 3T were not entirely the same as those acquired at 7T, we took a single slice at a similar level for each participant.

### Qualitative analysis

All images were viewed on 24-bit gray-scale. The histograms of SI within the GP were obtained, and the histogram metrics such as mean and standard deviation were recorded. The window settings (window width and window level) were adjusted to optimize visibility of the MML and the AML for the bilateral GPs of individual images using the histograms: the window level of the mean pixel value and the window width of ±3 × standard deviation for 3T-PDW, 3T-T2W, and 7T-PDW images and ±6 × standard deviation for 7T-T2W images were chosen. Two authors (S.M. and M.F.; 5 and 25 years of MRI experience, respectively) evaluated each image for the depiction of the MML and AML based on anatomical information from myelin staining in the atlas of Schaltenbrand and Wahren (Fig. 1)^16^. We assessed whether the MML and AML were visible by comparison with adjacent and surrounding tissues. The depiction of the MML and AML was determined as “visible” when more than half of them were delineated^35^. To decrease bias, the two authors resolved all disagreements by consensus reading of images. Particularly the images of an elderly volunteer were separately evaluated and were not included in the qualitative analysis of young volunteers.

### Quantitative analysis

Quantitative corrected SI maps were created using Interactive Data Language (IDL, Research Systems Inc., CA, USA) as previously described^38^ with minor modifications. In this study, we used low-resolution calibration data reconstructed from central k-space data for channel sensitivity estimation without additional scan data. The corrected SI of the root-sum-of-squares (RSS) of the combined signals of individual coil images was calculated as follows:

1 $$Corrected\,SI=\sqrt{{S}^{H}{\Psi }^{-1}S,}$$

where S denotes a vector containing the signals from an individual coil, and Ψ denotes the noise correlation matrix, which represents the noise statistics of the coils. Ψ can be calculated as follows:

2 $${\Psi }_{ij}=\sigma \cdot {\omega }^{2}\cdot {\int }_{V}{A}_{i}\cdot {A}_{j}dr,$$

where σ, ω, A, and V denote conductivity, resonance frequency, magnetic vector potential, and object volume, respectively, and *i*, *j* denote coil elements.

The average SI profiles were obtained at 0.5-mm intervals from the pixel value of the SI maps on a straight line perpendicular to the maximum diameter of the bilateral GP regions using ImageJ (version 1.8.0, National Institutes of Health, MD, USA). The SI profiles were normalized as a function of distance in the GP region using MATLAB R2018a (The MathWorks, Inc., MA, USA). We then averaged the SI profiles from each participant. This straight line was drawn manually by one author (S.M.) and validated by another author (M.F.) to confirm that the line did not include the blood vessels.

In order to quantitatively evaluate the variation of contrast in the GP region, we measured CRs based on the SI maps. For the MML, CRs were calculated between the GPe and MML and between the MML and the GPie. By contrast, for the AML, CRs between the GPie and AML and between the AML and GPii were calculated as follows:

3 $$CRs=\frac{S{I}_{j}}{S{I}_{i}},$$

where *i* = MML or AML and *j* = GPe, GPie, or GPii.

We calculated the average SIs in the MML and AML from three points around the negative peaks in the SI profiles, which were considered to correspond to the MML and AML because the thicknesses of the MML and AML are approximately 1 mm in axial slices^16^. We also measured average SIs in the GPe, GPie, and GPii from several points around the corresponding in the profile.

### Statistical analysis

All data are expressed as means ± standard deviation. Two-way analysis of variance (ANOVA) was performed on CRs with factors of the static magnetic field (3T, 7T) and sequence (PDW, T2W). A post-hoc two-sample t-test was performed when significant interaction effects were found. The Bonferroni multiple-comparison correction was performed to adjust the *P*-value. A *P* value less than 0.05 was considered to indicate statistical significance. All analyses were performed using the Statistical Package for the Social Sciences software (SPSS, version 25.0.0, IBM Corp., NY, USA).

## Data Availability

The datasets generated during the current study are available from the corresponding author on reasonable request.
